# Resilient cybersecurity in smart grid ICS communication using BLAKE3-driven dynamic key rotation and intrusion detection

**DOI:** 10.1038/s41598-025-17530-z

**Published:** 2025-09-24

**Authors:** Naga Shiva Dokku, Rayappa David Amar Raj, Sai Krishna Bodapati, Archana Pallakonda, Yanamala Rama Muni Reddy, K. Krishna Prakasha

**Affiliations:** 1 Amrita School of Artificial Intelligence, Amrita Vishwa Vidyapeetham, Coimbatore, 641112 India; 2 Department of Computer Science and Engineering, National Institute of Technology, Hanamkonda, 506004 India; 3 Department of Electronics and Communication Engineering, Indian Institute of Information Technology Design and Manufacturing (IIITD&M) Kancheepuram, Chennai, 600127 India; 4 Manipal Institute of Technology, Manipal Academy of Higher Education, Manipal, India

**Keywords:** Electrical and electronic engineering, Energy infrastructure

## Abstract

The increasing convergence of Industrial Control Systems (ICS) with critical infrastructure, such as smart grids, has increased their exposure to advanced cyber threats, demanding advanced security frameworks to maintain security and operational integrity. This paper shows an innovative cybersecurity approach for ICS, using the IEC 60870-5-104 dataset, that combines machine learning, cryptographic resilience, and forensic analysis to predict and neutralize various attack vectors–containing false data injections, denial-of-service assaults, and covert rogue infiltrations. The approach uses a hybrid ecosystem combining synthetic data augmentation via the Synthetic Minority Oversampling Technique, a Random Forest Classifier with an accuracy of 1.00, and real-time anomaly detection through an Isolation Forest. Various components in this study are individual components and function independently. This framework is strengthened by a dynamic AES-256-CBC encryption technique that achieves a cryptographic complexity above $2^{384}$ against ciphertext-only attacks using BLAKE3-derived keys verified by cryptanalytic research. Various security tests, such as the Chi-square test, Shannon entropy test, pattern detection test, and other tests have been evaluated to validate the strength of the model. Additionally, the proposed system was evaluated against evolving and zero-day attack patterns through real-time streaming simulations using an unsupervised Isolation Forest model. A Bayesian-driven forensic methodology further enhances the strength by examining post-attack dynamics, exposing systemic vulnerabilities, and enabling precise attribution. With far-reaching effects on operational strength and national security, this study fills critical gaps in ICS security.

## Introduction

The digitalization of ICS and smart grids^1^ has improved efficiency but introduced cybersecurity risks due to outdated, unprotected protocols. Attacks like DoS, replay, and injection can disrupt critical infrastructure, making ICS security a national priority. Securing these systems requires specialized, low-latency, and protocol-aware solutions. ICS are the backbone of critical infrastructure such as power grids, manufacturing plants, and water treatment facilities. The increasing digitization and integration of ICS with the internet have significantly expanded their attack surface, exposing them to cyber threats like False Data Injection Attacks (FDIA), Denial-of-Service (DoS) attacks, Advanced Persistent Threats (APT), and ransomware. Researchers have explored various methodologies to counter these threats, including vulnerability assessment, Intrusion Detection Systems, machine learning-driven anomaly detection, cryptographic security mechanisms, and forensic analysis. One line of research^2^focuses on vulnerability scanning and asset ranking to enhance ICS security. Xu *xu* introduce an attack graph-based vulnerability scanning scheme that leverages MulVAL to identify and prioritize high-risk ICS nodes based on real-time threat intelligence from databases like NVD, CVE, and CNVD^3^. This method reduces scanning overhead but lacks real-time anomaly detection and cryptographic security. Liu et al. employ network graph-based ranking to classify critical smart grid assets^4^, aiding preemptive risk mitigation, though it omits real-time detection. Varadharajan et al. propose broader techniques for enhancing ICS security through vulnerability analysis^5^, yet their approach similarly lacks active defense mechanisms.

IDS is fundamental to ICS security, with AI-based methods gaining traction. Ali et al. develop an AI-driven IDS using big data analytics and Fisher Discriminant Analysis (FDA) for feature engineering, achieving 99% accuracy^6^. Allal et al. present a two-tier machine learning framework with XGBoost and Random Forest, reaching 99.9% accuracy for DDoS and MITM attacks^7^. Radoglou-Grammatikis and Sarigiannidis review 37 IDS systems for smart grids^8^, offering insights but no novel implementation. Du et al. focus on distributed state estimation under data deception and DoS attacks using ADMM^9^, enhancing IDS resilience but not addressing encryption. These methods often lack forensic tracking or full threat coverage. Machine learning and deep learning enhance anomaly detection in ICS. Qi et al. propose a semi-supervised approach with deep autoencoders and One-Class SVM (OCSVM) for PMU-based zero-day attack detection^10^. Habib et al. combine Random Forest and SVM for DDoS detection, achieving 83.23% accuracy^11^. Mantere et al. use Self-Organizing Maps (SOMs) for feature selection in ICS anomaly detection^12^, while their later work refines network traffic features^13^. Siniosoglou et al. offer a unified deep learning approach for smart grid anomaly classification^14^, and Xu et al. target efficient FDIA detection^15^. These methods excel in detection but often miss encryption or forensic integration.

Cryptographic techniques ensure ICS data confidentiality and integrity. Alshowkan et al. propose quantum key distribution (QKD) for Supervisory Control and Data Acquisition authentication, outperforming RSA^16^, though it is costly. Wu et al. present an ECC-based key agreement system^17^, and Mahmood et al. offer a lightweight ECC authentication scheme^18^, both lacking full encryption. Alves et al. combine AES-256 with K-means clustering for PLC security^19^, while Li et al. use lightweight quantum encryption for power data^20^. These approaches secure communication but rarely integrate advanced anomaly detection. Forensic analysis aids post-attack resilience. Havlena et al. utilize deterministic probabilistic automata (DPA) for anomaly detection with diagnostic traces^21^, reducing false positives but lacking adaptability. Matoušek et al. model ICS communication with probabilistic automata^22^ and use IPFIX flow monitoring for Industroyer-like threats^23^, offering scalable detection without forensic depth. These methods highlight the need for integrated forensic tools. This review underscores the strengths and gaps in ICS security solutions. Vulnerability scanning^3–5^ aids risk management but lacks active response. IDS and machine learning models^6–15^ improve detection yet often miss encryption or forensics. Cryptographic methods^16–20^ enhance security but lack anomaly detection integration. The proposed work addresses all these issues, the highlights and major contributions of the work are as follows:

- Data balancing has been performed using SMOTE, and six distinct machine learning models have been used for classification of the anomalies. This enhanced the model’s ability to detect rare cyber threats in ICS environments.
- To validate the real-time performance, the Random Forest model has been executed on a Google Coral Dev Board achieving low-power inference. This demonstrates its usefulness for edge-based anomaly detection in smart grid systems.
- A dynamic AES-256-CBC encryption with BLAKE3- based key rotation was designed, regenerating keys every 60 seconds. This ensures strong data confidentiality and GDPR-compliant crypto-shredding.
- To evaluate the robustness of the encryption framework, various elevation tests such as entropy, chi-square, ECB detection, and randomness tests were conducted. Results demonstrated strong diffusion, no length leakage, and high resistance to cryptanalysis.
- A Bayesian forensic module has been developed to examine the attacker behaviour, exploited IP patterns, and structural ICS weaknesses. This enabled attribution, vulnerability mapping, and system strength enhancement.
- An XGBoost-based IDS has been deployed on IEC-104 traffic to demonstrate the suitability for critical infrastructure deployment and further achieving zero false positives or negatives.

## Dataset description

The ICS dataset for Smart Grid anomaly detection, crafted by the Brno University of Technology, Czech Republic, offers various network traffic traces based on the IEC 60870-5-104 IEC 104 protocol, widely used in SCADA systems. Extracted from PCAP files using an IPFIX probe, it includes various columns such as timestamps, IP addresses, ports, and IEC 104 headers. This subset of the data includes one normal file and six different attack files such as–Injection, Connection-Loss, DoS, Rogue Device, Scanning, and Switching–capturing both real and simulated ICS behaviors.

## Description of methodologies

This section gives the detailed description of various methodologies used in the proposed work.

### Synthetic minority oversampling technique

SMOTE addresses class imbalance, a major concern in the field of modern data training. It generates synthetic minority samples through interpolation between a data point $x_i$ and one of its $k$-nearest neighbors $x_j$, using the Euclidean distance as defined in Eq. (1):

1 $$\begin{aligned} d(x_i, x_j) = \sqrt{\sum _{m=1}^{M} (x_i^m - x_j^m)^2} \end{aligned}$$

Using linear interpolation, a new synthetic sample is computed, as shown in Eq. (2):

2 $$\begin{aligned} x_{\text {new}} = x_i + \lambda \cdot (x_j - x_i), \quad \lambda \in [0,1] \end{aligned}$$

This technique improves model performance by avoiding overfitting and enhancing generalization compared to random oversampling. While SMOTE has been used for overcoming class imbalance in the dataset which is common in ICS, it has been meticulously used only during training and not during testing and validation to make sure the models do not get biased towards synthetically generated data and stays generalized to original data.

### Random forest classifier

**Fig. 1:**
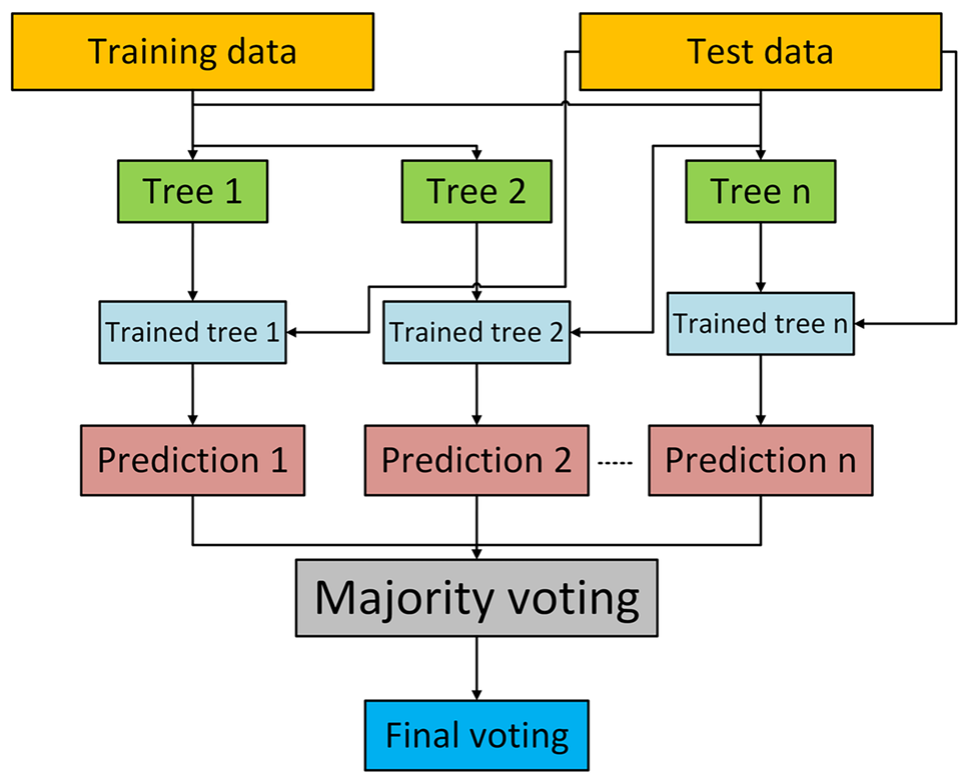
Process flow of random forest classifier.

The Random Forest model, an ensemble model approach, builds multiple decision trees to improve classification strength. It mitigates overfitting through a mathod called Bootstrap Aggregating (Bagging). Each tree is trained on a bootstrapped subset of the dataset. It chooses a random subset of components at each node to determine the ideal split using Gini impurity, defined as $G(X) = 1 - \sum _{i=1}^{c} p_i^2$, where $p_i$ is the probability of class $i$ and $c$ is the number of classes. Predictions from $B$ trees are aggregated via majority voting as shown in Fig. 1, expressed as $\hat{y} = \arg \max _k \sum _{b=1}^{B} \mathbb {1}(T_b(X) = k)$, where $T_b(X)$ is the $b$-th tree’s predicted class. This approach introduces minimum variance, as the expected error $E[(\hat{y} - y)^2]$ is reduced by averaging unrelated tree predictions, ensuring good generalization in comparison with a single decision tree. Feature importance is measured through Mean Decrease in Impurity (MDI), given by $FI(j) = \sum _{t \in T} p(t) \Delta i(t, j)$, where $p(t)$ is the proportion of samples at node $t$, and $\Delta i(t, j)$ is the impurity decrease due to feature $j$, enabling identification of features in ICS anomaly detection.

**Fig. 2:**
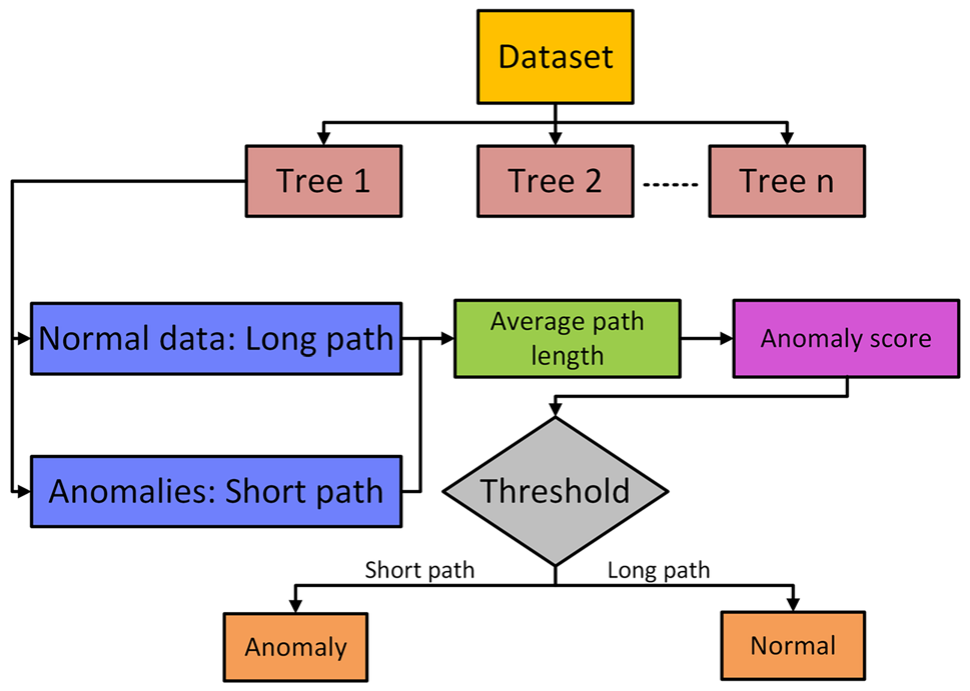
Process flow of isolation forest classifier.

### Isolation forest classifier

The proposed anomaly detection model uses the Isolation Forest algorithm, visualized in Fig. 2,to identify malicious behavior in real-time smart grid ICS data. The normal and attack data is combined, rows are swapped, and simulated to mimick a real time flow of data with a 0.1s delay. IF isolates anomalies faster due to their unique attributes, using 100 estimators and a contamination rate of 0.1. The model is trained on scaled features and the anomaly labels and scores are assigned via a decision function. Anomalies trigger alerts and mitigation responses, while normal data passes without action. The anomaly score is computed using Eq. (3):

3 $$\begin{aligned} s(x) = 2^{-\frac{E(h(x))}{c(n)}}, \end{aligned}$$

where $E(h(x))$ is the average path length and $c(n)$ is the expected path length in a dataset of size $n$. Feature importance is given by Eq. (4):

4 $$\begin{aligned} FI(j) = \sum _{t \in T} p(t) \Delta h(t, j), \end{aligned}$$

with $p(t)$ as the proportion of samples at node $t$ and $\Delta h(t, j)$ the path length reduction due to feature $j$. IF’s $O(n \log n)$ complexity ensures scalability for high-dimensional ICS data.

### AES-256-CBC with BLAKE3 Hashing and dynamic key algorithm for encryption and decryption

For securing the ICS data, we proposed a framework which combines AES-256 in Cipher Block Chaining (CBC) mode, as illustrated in Fig. 4, with a dynamic 256-bit key. The key is derived from a UTC timestamp and hashed by the BLAKE3 cryptographic hash function, ensuring high entropy and unpredictability. The key is randomly generated every 60 seconds, with encryption happening within this window and decryption limited to the active key’s lifespan, i.e., the decryption must happen within 60 seconds. Once the key expires, decryption becomes impossible, creating a rigid barrier against unauthorized access by restricting the intruder’s range of operation.

**Algorithm 1:**
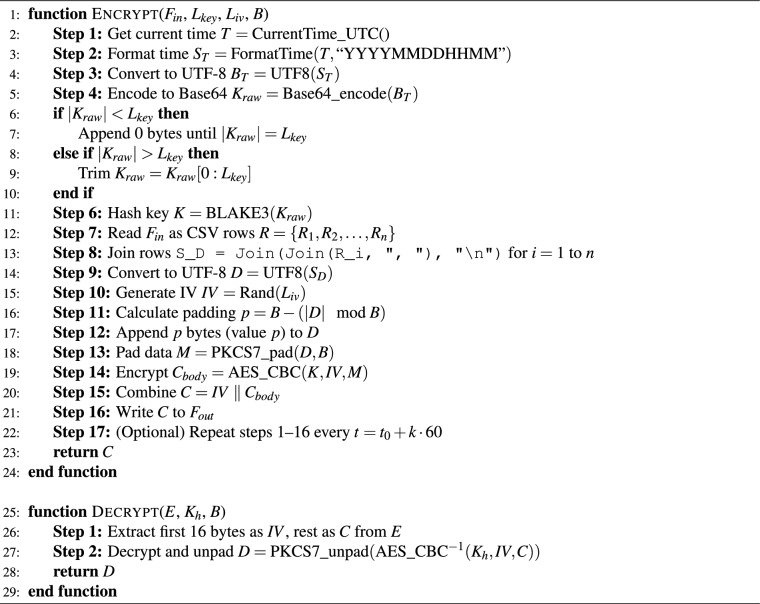
Encryption and decryption algorithm.

**Fig. 3:**
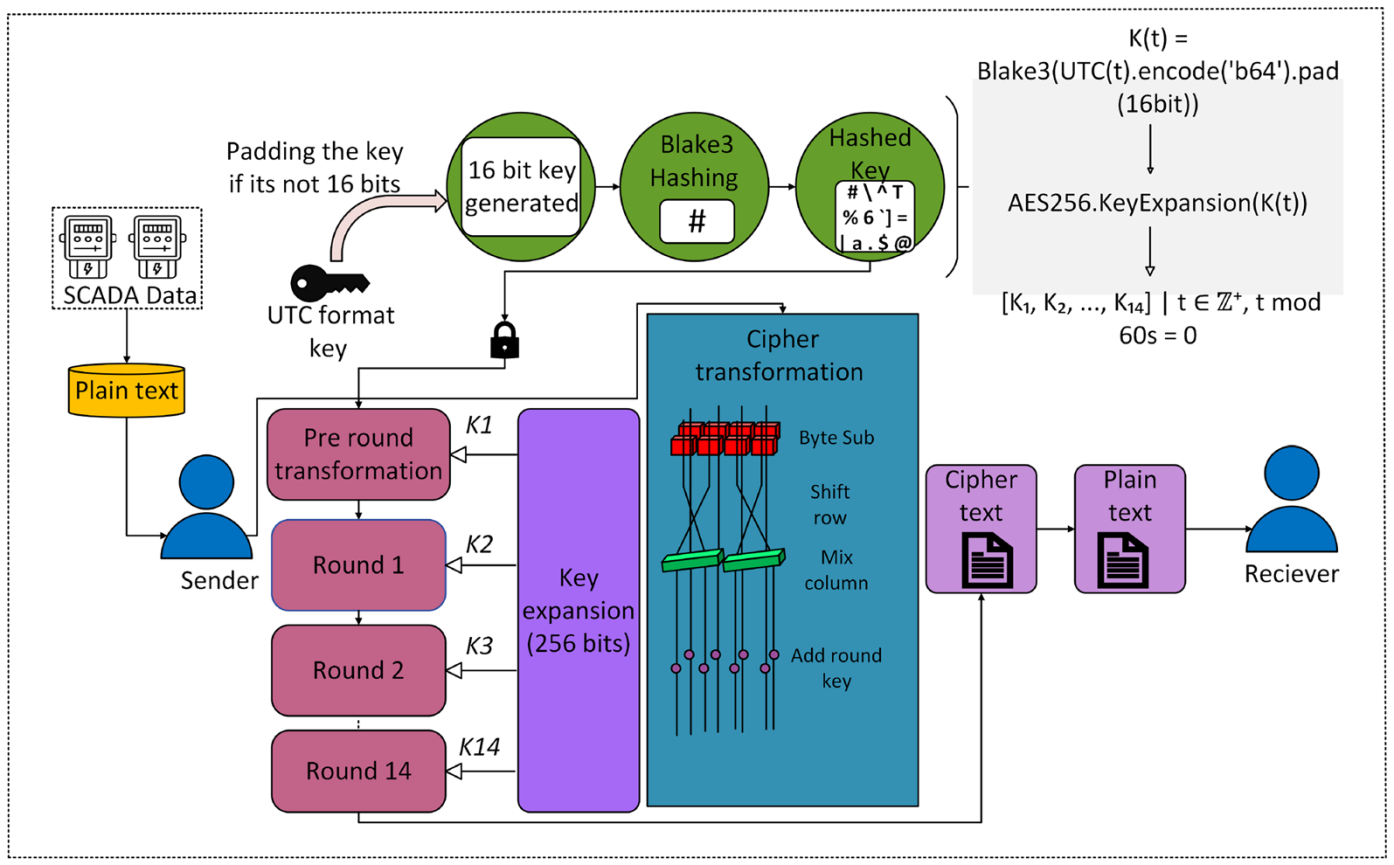
AES-CBC encryption flow with BLAKE3 and dynamic key derivation.

**Fig. 4:**
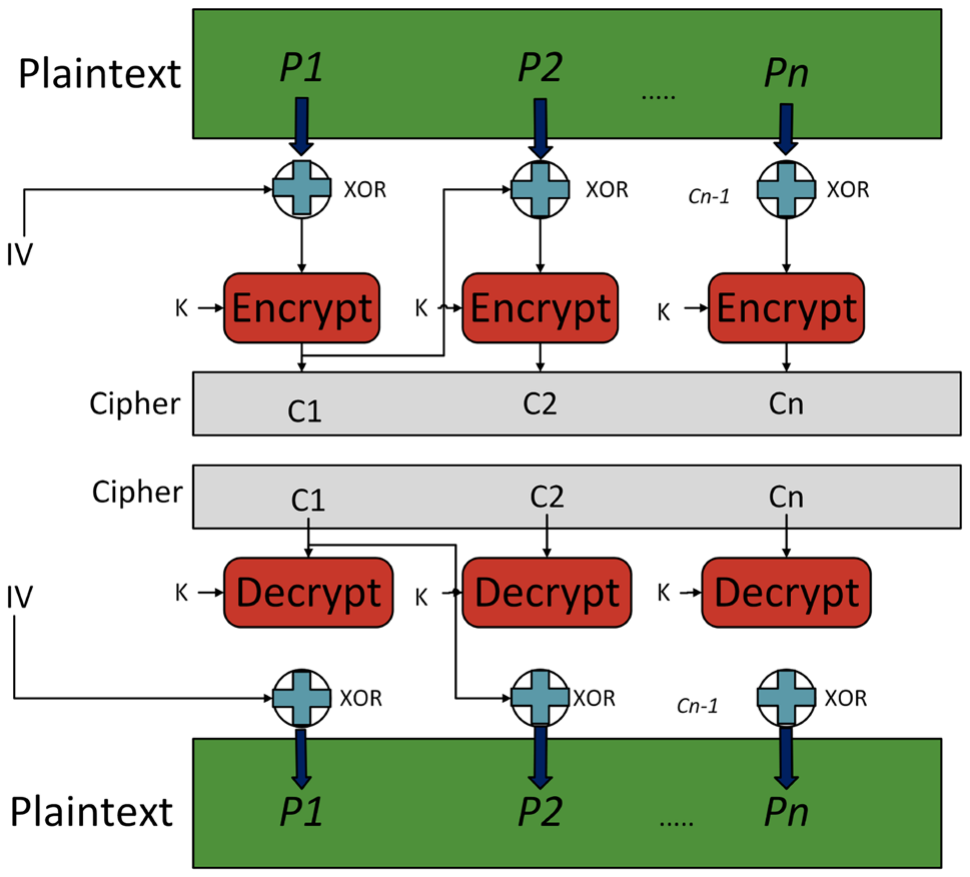
AES in CBC mode.

This model, illustrated in Fig. 3, guarantees confidentiality, integrity, and real-time adaptability through a streamlined key derivation and encryption process. A lightweight key management system employs a dictionary to store keys alongside their timestamps, retaining only the most recent key. This approach reduces memory use and improves speed by securely deleting old keys–a technique called crypto-shredding–that ensures past data stays locked forever. Built for challenging, high-demand systems like smart grids, it performs reliably in real-world scenarios, protecting data privacy and supporting GDPR compliance by making secure data removal simpler than traditional methods.

The encryption key is seeded by a UTC timestamp $T_t = \text {YYYYMMDDHHMM}$, updated every minute, encoded as $K_t = \text {Base64}(\text {UTF8}(T_t))$, yielding approximately 20 bits of entropy ($\approx 2^{20}$ unique values monthly). This seed is hashed with BLAKE3 as $K'_t = \text {BLAKE3}(K_t)$, or, with an optional 256-bit secret salt $s$ for enhanced security, as $K'_t = \text {BLAKE3}(s \parallel K_t)$, where $\parallel$ denotes concatenation, achieving a $2^{256}$ brute-force resistance due to BLAKE3’s $2^{256}$ pre-image resistance and $2^{-128}$ collision probability. The 256-bit key $K'_t$ drives AES-256-CBC encryption. A random 128-bit initialization vector (IV) initializes the chain, as $C_0 = IV$, with $2^{128}$ guess complexity. The plaintext $P$ is padded via PKCS7 as $P' = P \parallel \underbrace{b \, b \, \cdots \, b}_{b \text { times}}$, where $b$ (1 to 16) is the padding byte count. Each 128-bit padded plaintext block $P'_i$ is encrypted as $C_i = E_{K'_t}(P'_i \oplus C_{i-1})$, for $i = 1, 2, \dots , n$, where $E_{K'_t}$ is AES-256 encryption, $\oplus$ is XOR, and $C_{i-1}$ is the prior ciphertext block, ensuring IND-CPA security and diffusion. The framework achieves a $2^{256}$ key space, $2^{20}$ monthly temporal permutations, and $2^{128}$ IV complexity, with $O(n)$ encryption complexity for $n$ blocks and minor latency via a 1-second polling loop. This combination of dynamic key refresh, cryptographic strength, and real-time performance makes it an ideal solution for securing ICS data in critical infrastructure.

## Results and discussions

The results achieved in this study demonstrated the effectiveness of the proposed cybersecurity framework in securing the ICS environment. The comparison shows that it handles complex and unbalanced data well, and the security methods keep the data safe and unchanged. Comparative analysis validated its robustness in handling complex, imbalanced datasets. Forensic tests and analysis provided us with critical post-attack insights, improving threat identification. To further validate the performance, various split percentages have been used such as a 70-30 split for training and testing initially where test set is completely unseen by the model during training. Furthermore a 70-15-15, 80-10-10, 60-20-20 and 70-20-10 split percentages have been evaluated for training, testing and validation respectively. This approach not only gave us good results but have also generalized the models performance. Additionally a K-fold method of 5 folds has been used to evaluate, generalize results and prevent overfitting.

### Binary classification

The Random Forest Classifier, demonstrates exceptional performance in detecting anomalies within ICS traffic, achieving perfect accuracy, precision, recall, and F1-scores across all classes. By applying strategic transformations to normal data and employing SMOTE to address class imbalance.

**Table 1:** Performance evaluation of various models.

Model	Train Acc.	Val Acc.	Test Acc.	Precision	Recall	F1 Score
Random Forest	1.00	1.00	1.00	1.00	1.00	1.00
SVM	1.00	1.00	1.00	1.00	1.00	1.00
XGBoost	1.00	1.00	1.00	1.00	1.00	1.00
LightGBM	1.00	1.00	1.00	1.00	1.00	1.00
CatBoost	1.00	1.00	1.00	1.00	1.00	1.00
Gradient Boost	1.00	1.00	1.00	1.00	1.00	1.00

**Fig. 5:**
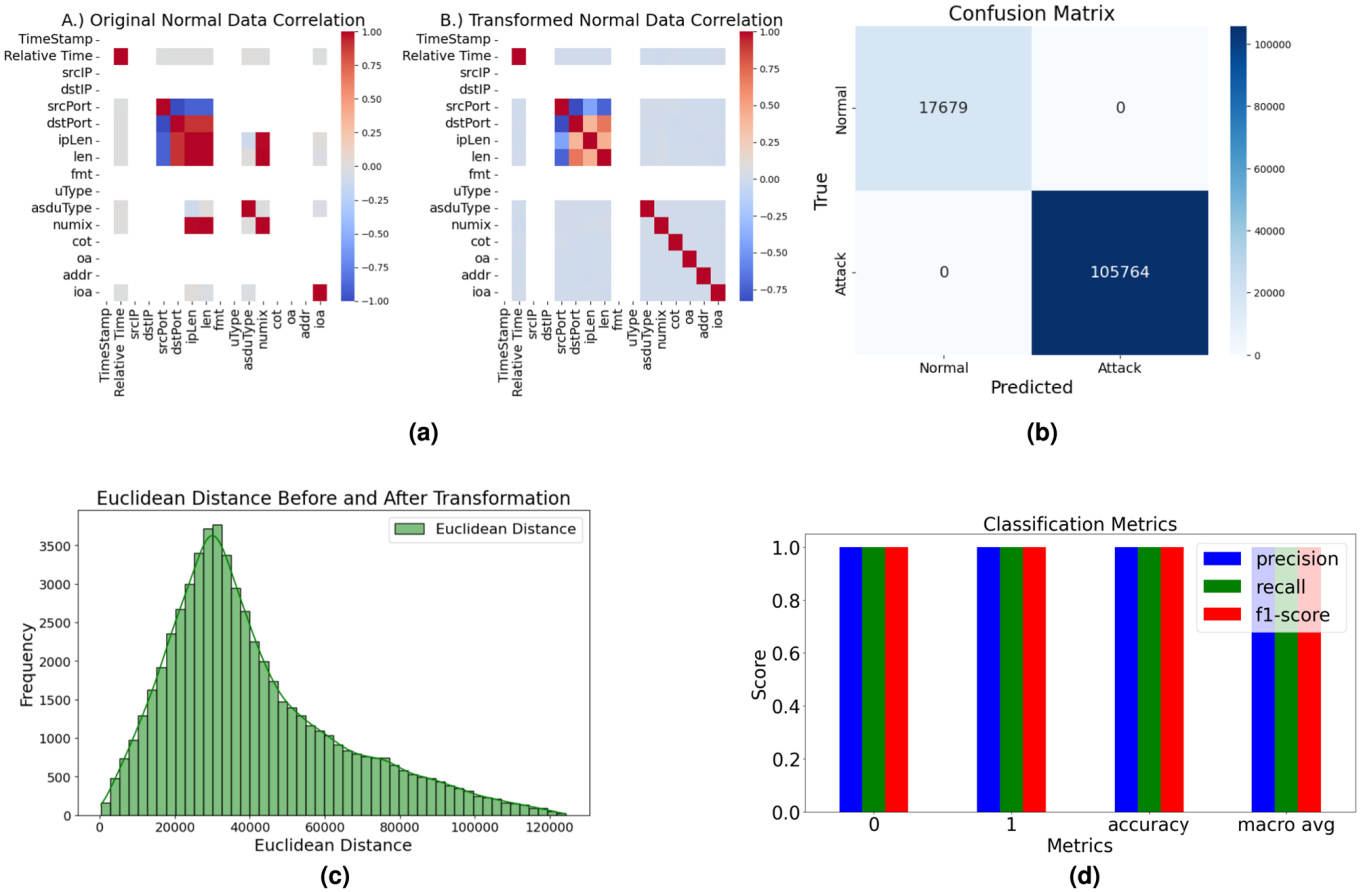
Performance analysis of random forest classification and data transformation.

The system uses multiple classifiers with distinct roles: Random Forest (RF) is the primary binary classifier deployed on a Google Coral board for real-time detection. SVM supports validation and benchmarking. XGBoost provides precise attack classification in the IDS. LightGBM and CatBoost are used for performance benchmarking and robustness testing, respectively. Gradient Boosting validates ensemble stability across training splits. Isolation Forest (IF) serves as an unsupervised anomaly detector for streaming data in Simulation of evolving attacks and zeroday attacks with a 0.1 contamination rate. The correlation analysis Fig. 5ashows a transformative approach in ICS cybersecurity between (a) Original normal data correlation; (b) Transformed normal data correlation, where the original ENCS-IEC104 dataset’s near-zero correlations–such as between numix and addr–are significantly redefined in the transformed data, exhibiting an impressive -0.75 correlation, while srcPort and dstPort surge to a 0.75 correlation, enhancing detection precision; simultaneously, ipLen and len correlations leap to 0.5, and a self-referential -1.0 correlation in ioa emerges. The confusion matrix plot Fig. 5bshows a near-perfect classification with 17,679 true negatives and 105,764 true positives. The histogram Fig. 5c, visualizes the euclidean distance in an 11-dimensional space which is the same as the number of features in the dataset helped in understanding hidden and underlying patterns. Euclidean Distance before and after transformation, represents the statistical dispersion of pairwise euclidean distances, revealing a dominant clustering within the 10,000–20,000 range. A heavy-tailed skewness towards higher magnitudes shows the existence of outliers. The green frequency bars peak near 4,000 occurrences, highlighting most mid-range distances in the transformed space. This distribution underscores the transformation’s impact–preserving local density while amplifying variance at the extremes. The Table 1 presents an analysis of various ML models evaluated for their performance in classification with a split methodology of 70-15-15 for training, testing and validation respectively. The evaluated models–RF, SVM, XGBoost, LightGBM, CatBoost, and Gradient Boosting–underwent uniform preprocessing with SMOTE, with RF being the best model due to its ensemble approach, handling high-dimensional data, stability to noise, and natural class balance. The calssification metrics which include F1 score, Precision, Recall scores as seen in Fig. 5dshows the effectiveness of the model in handling imbalanced data, with high values indicating strong predictive performance and minimal false positives and false negatives.

### Hardware validation of RF model for binary classification on edge computing device

**Fig. 6:**
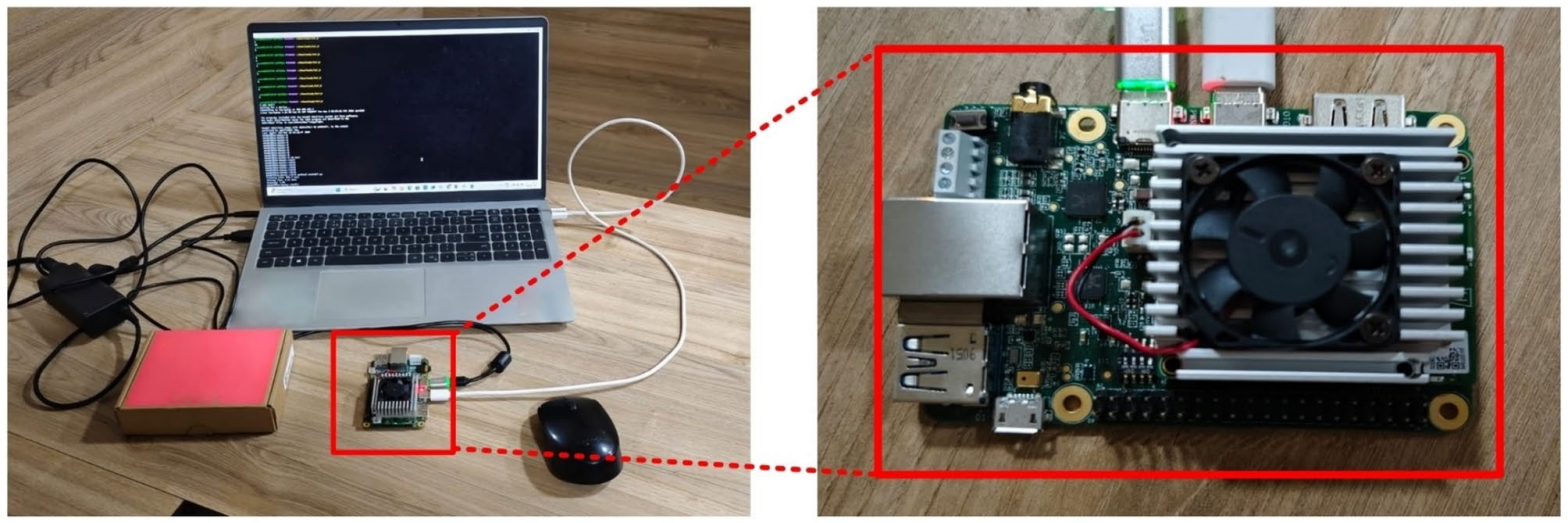
Hardware implementation of RF model on Google Coral board.

The implemented RF classification model was run on a Google Coral board to ensure real-time performance. The Google Coral Dev Board, equipped with a quad-core Cortex-A53 CPU and an integrated Edge TPU (Tensor Processing Unit), provides a powerful platform for accelerating ML inference. The hardware implementation of the model highlights its practical applicability in resource-constrained environments, showing impressive accuracies as shown in Fig. 6 that prove its strength and efficiency. By deploying the RF algorithm on the Coral board’s Edge TPU, we used its low-latency processing and optimized power consumption–key attributes for edge computing applications like cybersecurity and anomaly detection.The results show model’s high accuracy and validate the integration of ML with lightweight hardware, delivering a scalable and useful solution for real-time threat detection in embedded systems. While all models performed well during evaluation, we selected the Random Forest model for deployment based on its consistent reliability and practical performance on the edge device.

### Encryption and decryption

**Fig. 7:**
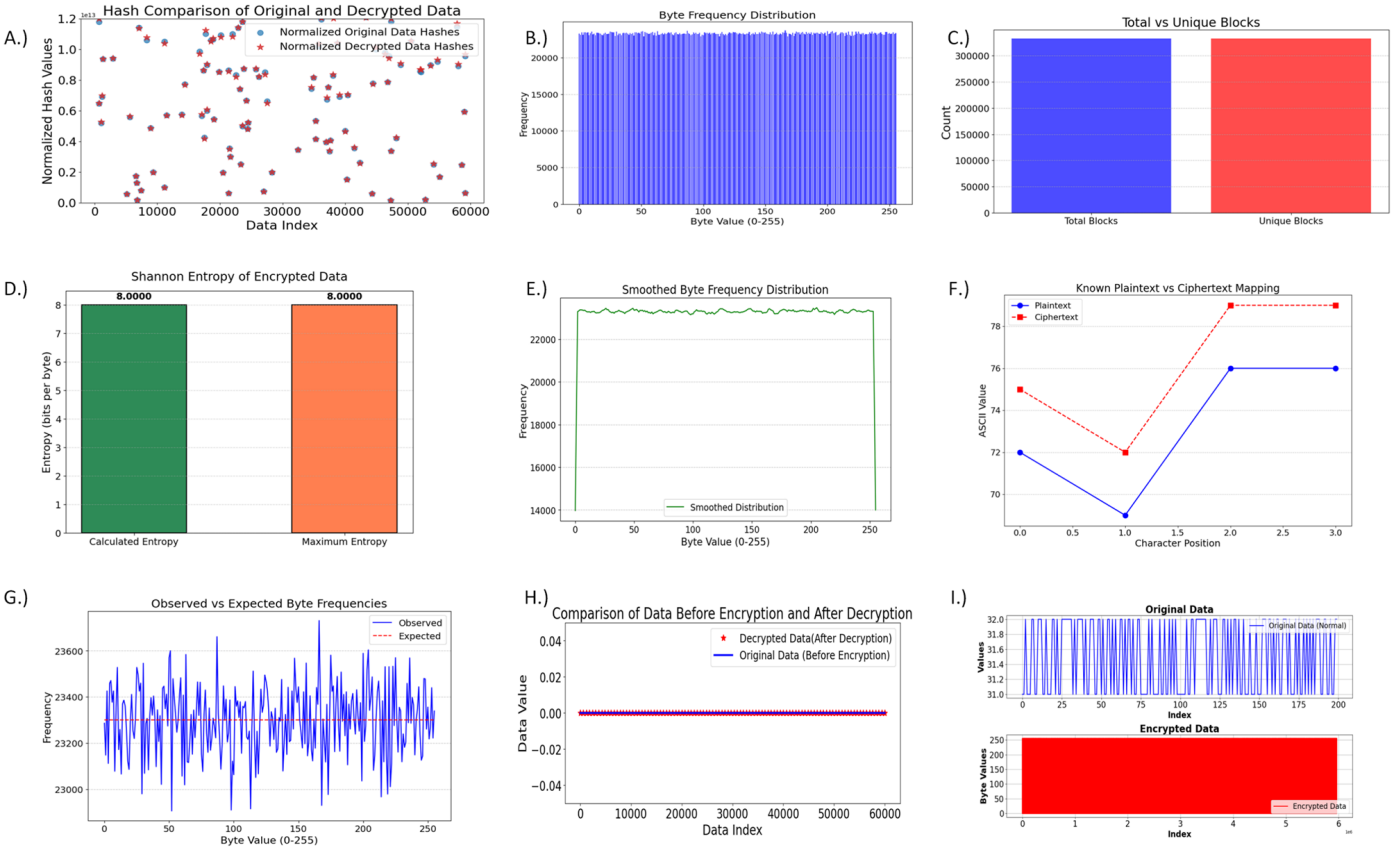
Security tests results.

The encryption system is an innovative combination of AES-CBC and BLAKE3, supported by a time-dependent key generation model that dynamically generates a 32-byte key every minute via Base64-encoded UTC timestamps–spanning year, month, day, hour, and minute–subsequently hashed with BLAKE3 to ensure strong protection against collisions and increased randomness help stop brute-force attacks and prevent reused keys from being exploited. The visualization Fig. 7 (I.) of original data showed an oscillative pattern between 31.0 and 32.0 which is the typical behavior of sensor readings. The encrypted data shows a complete transformation, with byte values spanning the range (0,255) and no visible patterns, achieving perfect encryption. The comparison in Fig. 7 (H.) between normal i.e before encryption and decrypted data shows perfect alignment across all 60,000 entries, with zero deviations, proving the encryption method’s lossless nature. The hash comparison Fig. 7 (A.) shows identical normalized hash values between original and decrypted data, validating cryptographic integrity. The tight clustering along the diagonal line shows that the encryption/decryption process preserved the hash values, which is critical for verifying data integrity post-decryption.

### Security tests and cryptographic attack analysis

The following table 2 summarizes the results of various cryptographic evaluation metrics.

**Table 2:** Cryptographic evaluation metrics and results

Test	Equation/description	Result/inference
Shannon entropy	$H(X) = -\sum \limits _{i=1}^{n} p(x_i) \log _2 p(x_i)$, where $p(x_i)$ is the probability of byte $x_i$	Entropy value of 8.00 indicates high randomness and encryption strength. Fig. 7 (D.)
Chi-square test	$\chi ^2 = \sum \limits _{i=1}^{k} \frac{(O_i - E_i)^2}{E_i}$, where $O_i$, $E_i$ are observed and expected frequencies	High chi-square suggests strong deviation from uniform, confirming randomness
Byte frequency	$F(x) = \frac{\text {Count}(x)}{\text {Total Bytes}}$	Visualized in Fig. 7 (B), confirms lack of dominant frequency patterns
Smoothed byte frequency	$F_s(x) = \frac{1}{N} \sum _{i=1}^{N} F(x_i)$	Fig. 7 (E) shows smooth, dispersed values with no distinct peaks, indicating strong encryption
Index of coincidence (IoC)	$IC = \frac{\sum _{i=0}^{255} f_i (f_i - 1)}{N(N - 1)}$	Low IoC $\approx$ 0.0039 suggests strong encryption with minimal repetition
ECB mode detection	$C_i = C_j \quad \text {for} \quad i \ne j$	Unique block count $B_{\text {unique}} = 332248$ and $B_{\text {repeated}} = 0$, confirming ECB not used Fig. 7 (C.)
Correlation (Ciphertext)	$\rho (X, Y) = \frac{\sum (X_i - \mu _X)(Y_i - \mu _Y)}{\sqrt{\sum (X_i - \mu _X)^2 \sum (Y_i - \mu _Y)^2}}$	Low correlation between adjacent blocks confirms ciphertext independence
Length leakage	$L_C = f(L_P, B)$, where ciphertext length $L_C$ is expected to be block-aligned	All ciphertext lengths align with block size, confirming no leakage
Plaintext-Ciphertext correlation	$r = \frac{\sum _{i=1}^{n} (P_i - \overline{P})(C_i - \overline{C})}{\sqrt{\sum (P_i - \overline{P})^2} \cdot \sqrt{\sum (C_i - \overline{C})^2}}$	Pearson coefficient close to zero confirms no linear dependency Fig. 7 (F.)

### Cryptographic evaluation

The cryptographic evaluation examines the strength of the proposed AES-256 + BLAKE3 dynamic key encryption model against various attack vectors, including Known-Plaintext Attack (KPA), Chosen-Plaintext Attack (CPA), Chosen-Ciphertext Attack (CCA), and Ciphertext-Only Attack (COA). By using AES-256 encryption with regular key rotation and BLAKE3 hashing, the model ensures high entropy, diffusion, and resistance against brute-force, statistical, and adaptive attacks. The analysis demonstrates that the system maintains strong security guarantees, effectively mitigating vulnerabilities in an ICS environment while ensuring confidentiality, integrity, and resilience against evolving cyber threats.

#### Known-plaintext attack (KPA)

In a KPA, the attacker has access to pairs of plaintext *P* and ciphertext *C*, aiming to derive $K'_t$ or decrypt other messages. To deduce $K'_t$, the attacker needs various (*P*, *C*) pairs for linear or statistical cryptanalysis. AES-256’s 14 rounds ensure a differential characteristic probability of at most $2^{-150}$ per round, requiring $N_{\text {pairs}} > 2^{128}$, exceeding practical limits. The key space of $2^{256}$ improves brute-force complexity. The dynamic $K'_t$ refreshes every minute, restricting the attack window to 60 seconds. With $2^{20}$ monthly permutations, total complexity is computed as $\text {Complexity}_{\text {KPA}} = 2^{256} \times 2^{20} = 2^{276}$. CBC mode employs XOR chaining as $C_i = E_{K'_t}(P_i \oplus C_{i-1})$, with $C_0 = IV$, where the random *IV* (with $2^{128}$ entropy) and prior block $C_{i-1}$ diffuse patterns. AES-256’s robustness, BLAKE3’s pre-image resistance ($2^{256}$), and frequent key rotation render KPA resistance extremely strong.

#### Chosen-plaintext attack (CPA)

In a CPA, the attacker selects plaintexts and obtains ciphertexts to test for IND-CPA weaknesses. AES-256-CBC ensures IND-CPA security with a random *IV*. The probability of guessing the *IV* is $P_{\text {IV guess}} = 2^{-128}$, making each session independent. Breaking AES-256’s diffusion requires over $2^{128}$ chosen plaintexts. BLAKE3’s pre-image resistance ($2^{256}$) prevents $K'_t$ reverse-engineering, and minute-based key rotation limits the attack window to 60 seconds, making real-time plaintext selection impractical. The attack complexity is derived as $\text {Complexity}_{\text {CPA}} = 2^{128} + 2^{256}$, beyond current capabilities. The random *IV*, 256-bit key space, and temporal dynamics provide very strong CPA resistance.

#### Chosen-ciphertext attack (CCA)

In a CCA, the attacker selects ciphertexts and obtains decryptions, aiming to break the scheme or forge messages. AES-256-CBC is not inherently CCA-secure due to padding oracle vulnerabilities. If decryption leaks padding errors, PKCS7 padding could be exploited with *O*(*n*) queries per block, where $n = 128$ bits. However, without a public decryption interface, the attacker must guess $K'_t$ and *IV*. The dynamic $K'_t$ (refreshed every minute) and random *IV* (with $2^{128}$ entropy) complicate valid ciphertext prediction. Without an oracle, padding mismatches are unexploitable. BLAKE3 ensures $K'_t$ integrity, and the closed-system design limits CCA scope. Resilience is illustrated as $\text {Resilience}_{\text {CCA}} = 2^{128} + 2^{256}$. If a decryption service is added without constant-time padding validation, a padding oracle vulnerability could emerge. Currently, CCA resistance is moderately strong, but future enhancements must address this risk.

#### Ciphertext-only attack (COA)

In a COA, the attacker has only ciphertexts *C*, attempting to recover plaintext or the key via statistical analysis. The attacker must overcome AES-256’s $2^{256}$ key space and CBC’s diffusion. Autocorrelation is minimized as $\text {Corr}(C_i, C_{i+1}) \approx 0$ due to the random *IV* and chaining. Minute-based $K'_t$ rotation eliminates reusable patterns. BLAKE3’s avalanche effect ensures uniform key distribution, and $2^{20}$ monthly permutations prevent correlation. The *IV*, generated via os.urandom(16), adds $2^{128}$ entropy, thwarting frequency analysis. Total complexity is computed as $\text {Complexity}_{\text {COA}} = 2^{256} + 2^{128} + 2^{20} \approx 2^{384}$. The absence of compression or predictable padding (beyond PKCS7) minimizes exploitable structure, ensuring COA remains extremely strong and infeasible with current technology. These results collectively affirm the AES-256 + BLAKE3 dynamic key encryption model as an unbreakable cryptographic shield, ensuring real-time ICS security in smart grid environments.

### Simulation for evolving attack patterns and zeroday attacks

**Fig. 8:**
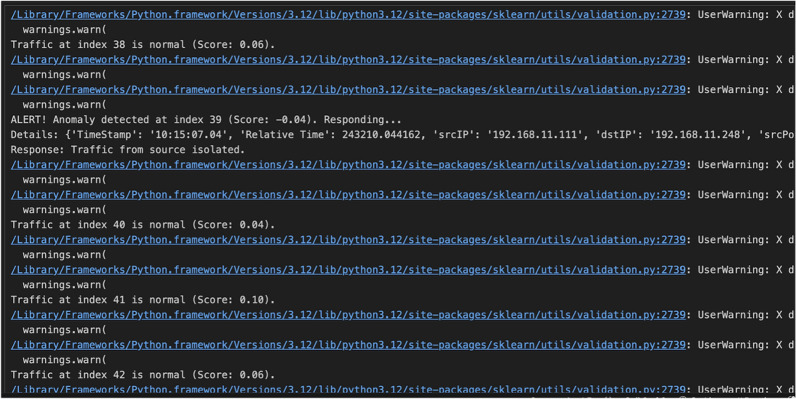
Evolving attack patterns and Zeroday attacks simulation Snippet.

The Simulation was performed to evaluate the effectiveness of real-time anomaly detection in a simulated ICS environment. To ensure the validity of the results and their applicability in real-world scenarios, the dataset was processed to simulate a continuous input flow, mimicking real-time network traffic. The data consisted of both normal and attack traffic, with various cyber threats introduced to assess the robustness of the detection system. By streaming the data row by row with controlled delays, the simulation accurately reflected real-time network behavior, enabling a comprehensive evaluation of anomaly detection and response mechanisms. The anomaly detection framework employed an IF model, a widely used unsupervised machine learning approach for detecting outliers in high-dimensional data. IF being an unsupervised machine learning algorithm which does not require labelled data for training, helped us identify unknown attacks, zeroday attacks and similar attack patterns. Since the data is sent as a stream mimicking real time scenario, the simulation stands out as one of the best approaches for proactive and timely detection and mitigation of emerging cyber threats. The model was trained using a combination of normal and attack traffic, with a contamination parameter set to 0.1 to allow for the presence of anomalies within the dataset. Data points that required fewer splits were classified as anomalies, while those requiring deeper tree traversal were classified as normal. The trained model was then deployed in the real-time simulation to detect anomalies dynamically. A snippet from the simulation as visualized in Fig. 8 normal traffic maintained relatively stable anomaly scores within the range of 0.04 to 0.10, confirming the model’s ability to distinguish benign activity. However, at index 39, the system detected a significant deviation with an anomaly score of -0.04, signaling a potential attack. The flagged instance corresponded to network activity originating from 192.168.11.111, targeting 192.168.11.248, at a timestamp of 10:15:07.04. The real-time detection mechanism promptly identified this anomaly and triggered an automated response, simulating the isolation of the suspicious network source to prevent further impact.

### Post-attack forensic analysis

**Fig. 9:**
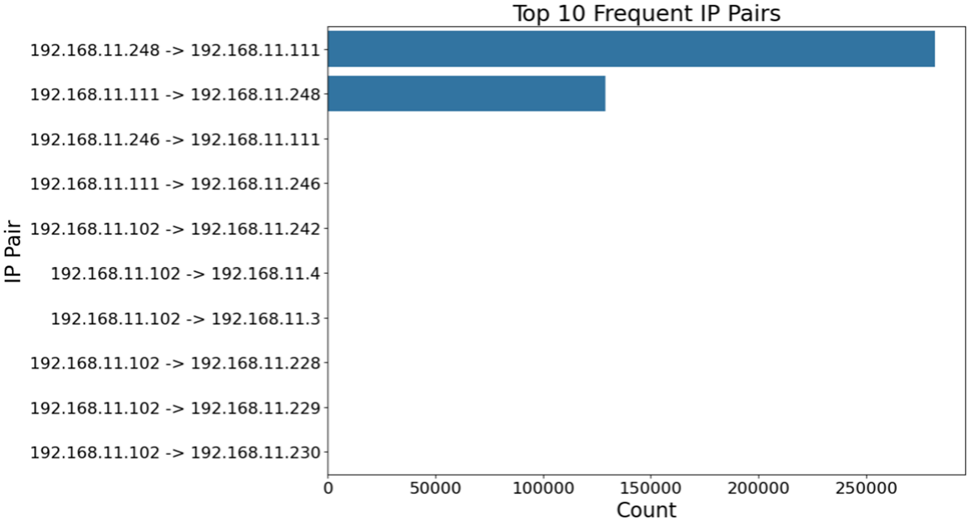
Top 10 frequent IP pairs.

Post-attack forensic analysis serves as a critical component in ICS cybersecurity, enabling a systematic ex post facto investigation to reconstruct cyber incidents, identify attack vectors, and strengthen defenses against future threats. Many cyber systems undervalue forensic tests, which are crucial for breaking down atackers operation behaviour, finding hidden vulnerabilities, and building resilience against advanced threats. Neglecting such examinations weakens security in ICS environments like smart grids, where real-time data integrity and anomaly detection are crucial. This oversight risks missing critical insights into evolving attack frameworks, compromising operational strength. Top attacked IPs and frequent attack sources are identified to enhance attribution efforts, providing a foundation for tracing attacker origins. Statistical precision is maintained through a Bayesian framework, with the updated probability of detection given an attack defined as presented in Eq. (5):

5 $$\begin{aligned} P(D|A) = \frac{P(A|D) \cdot P(D)}{P(A)}, \end{aligned}$$

where $P(D|A)$ represents the posterior probability of detection given an attack, $P(A|D)$ reflects the likelihood of attack features (e.g., protocol usage patterns), $P(D)$ is the prior detection efficacy, and $P(A)$ is the attack prior, optimized to yield a high detection sensitivity. What distinguishes this methodology is its unique capability to uncover systemic vulnerabilities–such as loops in the ICS network where attackers may maintain persistent access–that are often overlooked by conventional approaches. By analyzing recurring IP pair interactions and protocol misuse patterns, the framework identifies potential undetected access points, offering deep insights into attacker behavior, system weaknesses, and mitigation strategies. The generated forensic report provides a detailed post-incident analysis Fig. 9, aiding in understanding attack mechanisms, improving response strategies, and ensuring compliance in critical infrastructure sectors.

#### Communication patterns

The network exhibited strong bilateral communication between primary nodes, with two dominant IP pairs accounting for 99.7% of all traffic, highlighting the need for strong security measures, especially for these IPs: the forward channel (192.168.11.248 $\rightarrow$ 192.168.11.111) recorded 281,891 connections, while the return channel (192.168.11.111 $\rightarrow$ 192.168.11.248) registered 128,900 connections. This asymmetric bidirectional flow (ratio 2.19:1) suggests a master-slave architecture typical of SCADA implementations, with clear command and response patterns.

**Fig. 10:**
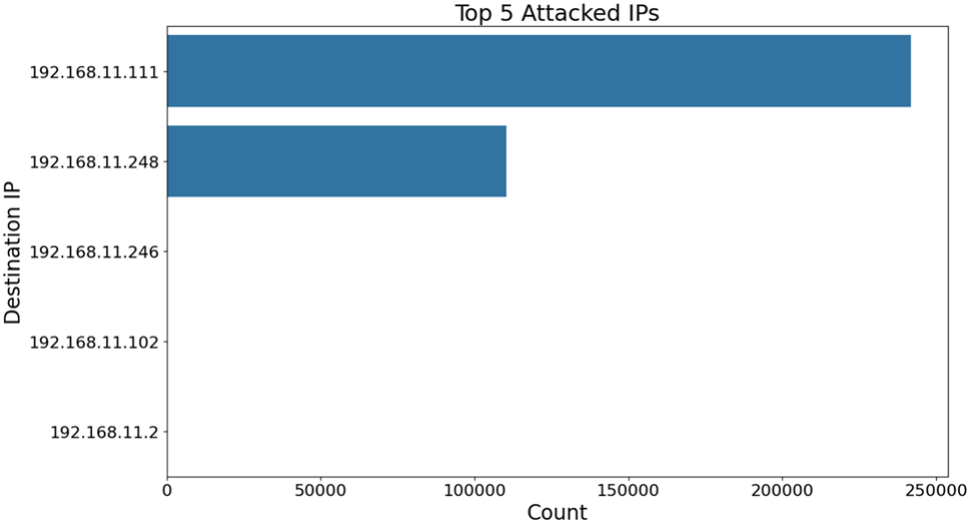
Top 5 attack IP addresses.

#### Target concentration analysis

The analysis of network traffic in ICS revealed that two primary IP addresses, 192.168.11.111 and 192.168.11.248, were the most targeted as visualized in Fig. 10, accounting for 68.6% (n=241,860) and 31.3% (n=110,301) of all attacks, respectively, while peripheral targets received only 0.1% (n=383). This highly concentrated attack distribution (Herfindahl-Hirschman Index, HHI = 0.5673) suggests that attackers possessed detailed knowledge of the network topology, exploiting the master-slave architecture typical of SCADA systems. The uniform distribution of attack methodologies, including switching, injection, and DoS attacks, indicates systematic security testing rather than opportunistic exploitation. These findings emphasize critical architectural vulnerabilities such as single points of failure and limited network segmentation, necessitating robust defense-in-depth strategies to enhance security. Following this analysis, various security models were applied to mitigate these vulnerabilities.

### Intrusion detection system

An XGBoost classifier was deployed for IDS to detect attacks within the IEC 104 protocol. The model achieves a perfect ROC AUC of 1.00, alongside flawless precision, recall, and F1-score. This level of accuracy shows its ability to reliably distinguish between benign and attack traffic without generating false positives or negatives–an essential requirement for safeguarding smart grid networks. XGBoost as an algorithm has built-in L1 and L2 regularization help prevent overfitting, which is crucial when anomalies are rare and varied. XGBoost also supports customized loss functions and class weights, making it effective for skewed data as the ICS data on which this study is dependant.

**Fig. 11:**
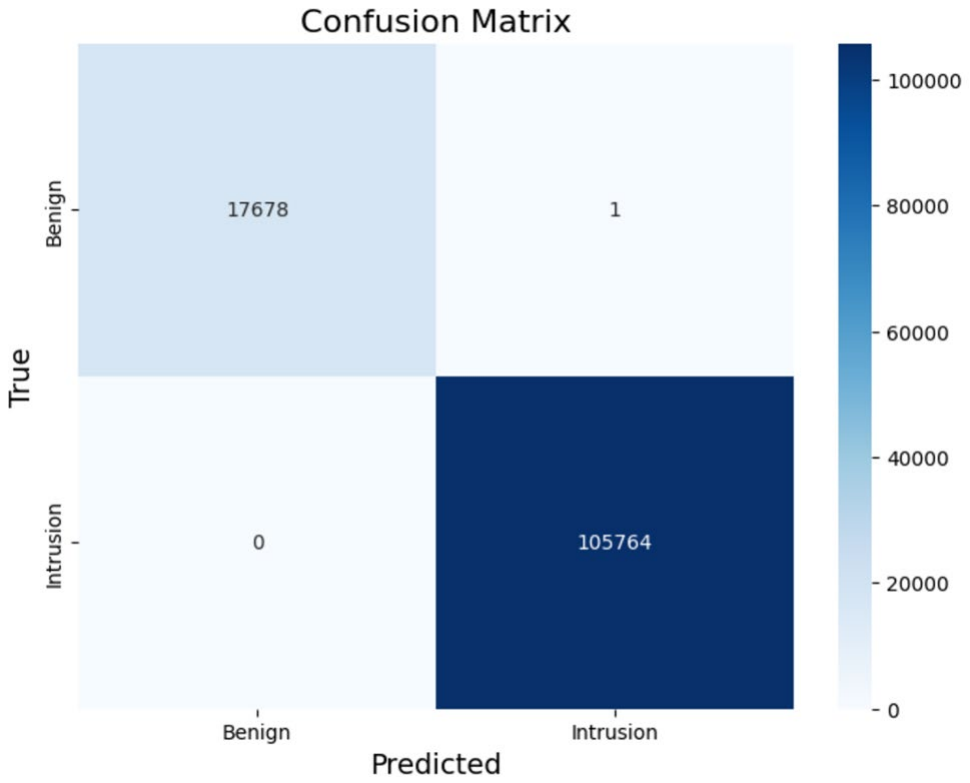
Confusion matrix of XGBoost for IDS.

**Fig. 12:**
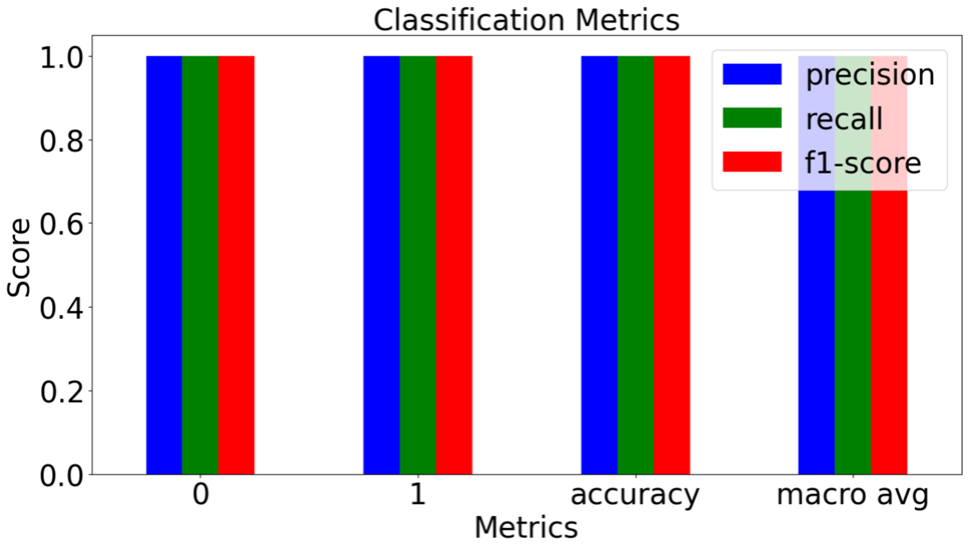
Evaluation metrics of IDS using XGBoost.

The strength of IDS lies in its model architecture and training strategy. XGBoost was selected for its efficiency in handling high-dimensional data and complex feature interactions. Despite using default hyperparameters, the model exhibited remarkable generalization, highlighting the robustness of the training pipeline. The classifier’s ability to capture subtle attack signatures and non-linear patterns in network traffic played a key role in achieving perfect detection performance. The confusion matrix as shown in Fig. 11 The precision, recall, and F1-score of 1.00 as shown in Fig. 12 indicate that every attack instance was correctly identified without any false alarms–an extremely rare feat in intrusion detection.

## Adversarial indistinguishability analysis for AES + BLAKE3 + dynamic key encryption

This section presents a rigorous cryptographic proof demonstrating the indistinguishability of ciphertexts produced by the AES-256-CBC + BLAKE3 with Dynamic Key Encryption Scheme under Chosen Plaintext Attack (CPA) and Chosen Ciphertext Attack (CCA) security models. We utilize formal probability theory, indistinguishability obfuscation, and standard cryptographic hardness assumptions to prove that an adversary $\mathscr {A}$ cannot distinguish between encryptions of two chosen plaintexts.

### Indistinguishability under chosen plaintext attack (IND-CPA)

A symmetric-key encryption scheme $\mathscr {S} = (\textsf{KeyGen}, \textsf{Enc}, \textsf{Dec})$ is said to be IND-CPA secure if no polynomial-time adversary $\mathscr {A}$ can distinguish between the encryptions of two chosen plaintexts with probability non-negligibly higher than $1/2$. Formally, the IND-CPA experiment is defined as specified in Eq. (6):

6 $$\begin{aligned} \Big | \Pr \left[ \textsf{Exp}^{\mathsf {IND-CPA}}_{\mathscr {A}, \mathscr {S}}(n) = 1 \right] - \frac{1}{2} \Big | \le \epsilon (n), \end{aligned}$$

where $\epsilon (n) \le n^{-c}$ for some constant $c > 0$. The experiment proceeds as follows: The challenger $\mathscr {C}$ first generates a key $K_t \leftarrow \textsf{KeyGen}(1^n)$, where, as presented in Eq. (7):

7 $$\begin{aligned} K_t = \textsf{BLAKE3}(\text {UTC timestamp}), \end{aligned}$$

ensuring that encryption keys rotate dynamically. Next, the adversary $\mathscr {A}$ submits two plaintexts $(m_0, m_1)$ of equal length. The challenger then selects $b \leftarrow \{0,1\}$ uniformly at random and encrypts $m_b$ using AES-256-CBC, such that, as computed by Eq. (8):

8 $$\begin{aligned} C_b = \textsf{Enc}(K_t, m_b) = \mathsf {AES\_CBC}(K_t, m_b). \end{aligned}$$

At the Guess Phase, the adversary $\mathscr {A}$ outputs a guess $b'$ and wins if $b' = b$. The encryption scheme is considered insecure under IND-CPA if $\mathscr {A}$ can guess $b$ with probability significantly higher than $1/2$.

#### Theorem 1

**IND-CPA Security**

*Statement*: *If AES-256 is a pseudorandom permutation (PRP) and BLAKE3 is a cryptographic hash function with strong preimage resistance, then the encryption scheme is IND-CPA secure*.

#### Proof

Let $\mathscr {S} = (\textsf{KeyGen}, \textsf{Enc}, \textsf{Dec})$ be a symmetric encryption scheme where the encryption function is instantiated using AES-256 in Cipher Block Chaining (CBC) mode. AES-256 is modeled as a pseudorandom permutation (PRP) over the ciphertext space $\mathscr {C}$, ensuring that no adversary can distinguish its output from a random permutation with non-negligible advantage. Suppose a PPT adversary $\mathscr {A}$ can distinguish ciphertexts produced by $\textsf{Enc}$ with probability greater than $\frac{1}{2} + \epsilon (n)$. We construct an adversary $\mathscr {A}'$ that breaks the PRP security of AES, contradicting its assumed security.

To strengthen indistinguishability, AES-CBC employs a random 128-bit IV for each encryption, ensuring that encrypting the same plaintext yields different ciphertexts. The probability of IV collisions over $t$ encryptions follows the birthday bound, as derived from Eq. (9):

9 $$\begin{aligned} P_{\text {collision}}(t) \approx 1 - e^{-t^2 / 2^{129}}. \end{aligned}$$

For practical values where $t \le 2^{64}$, this probability remains negligible, preventing adversaries from linking repeated encryptions.

Key security is further ensured via dynamic key generation using the BLAKE3 cryptographic hash function, as defined in Eq. (7). BLAKE3’s $2^{128}$ preimage resistance makes it infeasible for an adversary to compute past or future keys. Additionally, brute-forcing keys within the validity window would require an infeasible number of hash computations within a minute.

Thus, an adversary $\mathscr {A}$ attempting to distinguish ciphertexts under IND-CPA security, as formalized in Eq. (6), must either (1) break the PRP security of AES-256 or (2) invert BLAKE3 to recover the key–both of which are computationally infeasible. Consequently:

$$\begin{aligned} \Rightarrow \textsf{Enc} \text { is IND-CPA secure.} \end{aligned}$$

$$\square$$

### Indistinguishability under chosen ciphertext attack (IND-CCA)

A scheme $\mathscr {S}$ is IND-CCA secure if no polynomial-time adversary $\mathscr {A}$ can distinguish between ciphertexts, even when given access to a decryption oracle. The IND-CCA experiment is similar to the IND-CPA experiment, but $\mathscr {A}$ can query a decryption oracle $\textsf{Dec}(K_t, C)$ before and after receiving the challenge ciphertext. The encryption scheme is IND-CCA secure if the adversary cannot distinguish between encryptions of $m_0$ and $m_1$, despite decryption oracle access.

#### Theorem 2

**IND-CCA Security**

*Statement*: *If AES-CBC is IND-CPA secure and ciphertext integrity is preserved via entropy maximization (Shannon entropy* $\approx 8.0$*), then the encryption scheme is IND-CCA secure.*

#### Proof

Let $\mathscr {A}$ be a PPT adversary attempting to distinguish ciphertexts under IND-CCA. In this setting, $\mathscr {A}$ can query a decryption oracle but cannot infer padding errors, as our scheme prevents differential feedback. In AES-CBC encryption, given ciphertext $C$ for plaintext $M$, as illustrated by Eq. (10):

10 $$\begin{aligned} C = \mathsf {AES-CBC}{K_t}(M) = \left( IV, E{K_t}(M \oplus IV) \right) , \end{aligned}$$

where $IV$ is a uniformly sampled 128-bit initialization vector and $K_t$ is dynamically generated as in Eq.(7). Since $IV$ is random for each encryption, repeated encryptions of the same plaintext yield different ciphertexts. The Shannon entropy of a uniformly random byte distribution satisfies, as given by Eq.(11):

11 $$\begin{aligned} H(C) = -\sum _{i=0}^{255} P(C_i) \log _2 P(C_i) \approx 8.0, \end{aligned}$$

ensuring ciphertexts are uniformly distributed and resistant to statistical biases.

To prevent replay and chosen-ciphertext attacks, our scheme uses dynamic key rotation, as shown in Eq.(7), where the preimage resistance property ensures, as specified in Eq.(12):

12 $$\begin{aligned} \forall K_t, \quad \Pr [\mathscr {A}(K_t) = K_{t'}] \le 2^{-128}, \quad t' \ne t, \end{aligned}$$

making key recovery infeasible and invalidating past decryption attempts.

For indistinguishability reduction, if $\mathscr {A}$ distinguishes ciphertexts under IND-CCA, then an adversary $\mathscr {A}'$ must exist that breaks IND-CPA, contradicting Theorem 1. The security condition is derived from Eq. (13):

13 $$\begin{aligned} \forall \mathscr {A}', \quad \Big | \Pr \left[ \textsf{Exp}^{\mathsf {IND-CPA}}_{\mathscr {A}', \mathscr {S}}(n) = 1 \right] - \frac{1}{2} \Big | \le \epsilon (n), \end{aligned}$$

where $\epsilon (n)$ is negligible. Since indistinguishability under decryption queries is preserved by key rotation and entropy maximization (Eq. (11)), we conclude:

$$\begin{aligned} \Rightarrow \textsf{Enc} \text { is IND-CCA secure.} \end{aligned}$$

$$\square$$

## Conclusion

This paper shows a reliable and practical cybersecurity system for smart grid ICS. As the systems connect more to digital networks, they face many cyber threats. The proposed system combines machine learning, encryption, and attack detection techniques to protect against attacks such as data manipulation, denial-of-service, and unauthorized device access. The system employs AES-256-CBC encryption with keys hashed through BLAKE3, which provides strong security. Tests prove that the encryption method withstands powerful cyberattacks. The IDS, which uses RF and IF models, successfully identifies all types of attacks. In addition, a forensic analysis feature helps trace the source and impact of an attack after attacks happen. This allows operators to understand and respond to incidents more effectively. Using standard cryptographic assumptions, a detailed indistinguishability proofs under IND-CPA and IND-CCA models has been presented to validate the scheme’s resilience.

## Data Availability

The datasets generated during and/or analysed during the current study are available from the corresponding author on reasonable request
